# Insulin resistance and subclinical abnormalities of global and regional left ventricular function in patients with aortic valve sclerosis

**DOI:** 10.1186/1475-2840-13-86

**Published:** 2014-04-27

**Authors:** Hiroto Utsunomiya, Hideya Yamamoto, Eiji Kunita, Takayuki Hidaka, Yasuki Kihara

**Affiliations:** 1 Department of Cardiovascular Medicine, Hiroshima University Graduate School of Biomedical and Health Sciences, Hiroshima, Japan

**Keywords:** Aortic valve sclerosis, Cardiac function, Insulin resistance, Speckle-tracking echocardiography, Visceral adipose tissue

## Abstract

**Background:**

Insulin resistance, as a key mediator of metabolic syndrome, is thought to be associated with pathogenesis of calcific aortic valve disease and altered left ventricular (LV) function and structure. However, in patients with aortic valve sclerosis (AVS), the association between insulin resistance and subclinical impairment of LV function is not fully elucidated.

**Methods:**

We studied 57 patients (mean age 70 ± 8 years, 22 women) with asymptomatic AVS but normal LV ejection fraction in echocardiography. LV longitudinal and circumferential strain and strain rate was analyzed using two-dimensional speckle tracking echocardiography. Patients with uncontrolled hypertension and diabetes mellitus, chronic kidney disease, and concomitant coronary artery disease were excluded. They were divided into the insulin-resistant group (AVS+IR; N = 28) and no insulin-resistant group (AVS-IR; N = 29) according to the median value of homeostatic model assessment index. Computed tomography scans were also performed to measure the aortic valve calcium score and the visceral adipose tissue (VAT) area. In addition, age- and sex- adjusted 28 control subjects were recruited for the comparison.

**Results:**

There were no significant differences in LV ejection fraction or mass index among the groups. The AVS+IR group had a higher aortic valve calcium score (median 94 versus 21, *P* = 0.022) and a larger VAT area (113 ± 42 cm^2^ versus 77 ± 38 cm^2^, *P* = 0.001) than the AVS-IR group. Notably, LV global longitudinal strain, strain rate (SR), and early diastolic SR were significantly lower in the AVS+IR group than in the AVS-IR group and in control subjects (strain: -16.2 ± 1.6% versus -17.2 ± 1.2% and -18.9 ± 0.8%; SR: -1.18 ± 0.26 s^-1^ versus -1.32 ± 0.21 s^-1^ and -1.52 ± 0.08 s^-1^; early diastolic SR: -1.09 ± 0.23 s^-1^ versus -1.23 ± 0.18 s^-1^ and -1.35 ± 0.12 s^-1^; *P* < 0.05 for all comparison), whereas circumferential function were not significantly different. Multiple linear regression analyses revealed insulin resistance as an independent determinant of LV longitudinal strain (*P* = 0.017), SR (*P* = 0.047), and early diastolic SR (*P* = 0.049) regardless of LV mass index or VAT area.

**Conclusions:**

Insulin resistance is a powerful independent predictor of subclinical LV dysfunction regardless of concomitant visceral obesity and LV hypertrophy. Thus, it may be a novel therapeutic target to prevent subsequent heart failure in patients with AVS.

## Background

Aortic valve sclerosis (AVS) is a common echocardiographic finding in the elderly and is defined as calcified and thickened aortic leaflets without restriction of leaflet motion [1]. This is in contrast to aortic valve stenosis, in which the motion of calcified and thickened leaflets is restricted. AVS is associated with increased cardiovascular risk [2] and subclinical atherosclerosis [3] leading to cardiovascular mortality and morbidity [4]. Interestingly, AVS increases the risk of developing congestive heart failure even in the absence of hemodynamically significant obstruction of left ventricular (LV) outflow [1].

The constellation of conditions comprising a dysmetabolic state, such as insulin resistance (IR), excess visceral adiposity, dyslipidemia, and proinflammation, is often referred to as *metabolic syndrome*[5]. In the previous clinical trial, in addition to compensatory LV hypertrophy caused by pressure overload, IR was a powerful independent predictor of progression of LV hypertrophy in patients with calcific aortic valve stenosis [6]. In addition, several studies indicate that both IR and visceral obesity are associated with not only the incidence of AVS [7] but also impaired LV systolic and diastolic function even after accounting for comorbidities such as coronary artery disease [8,9]. However, the association between IR and an impairment of LV function in AVS remains unclear.

To date, LV ejection fraction is the most commonly used method to assess myocardial function. However, it is not a sensitive measure of systolic dysfunction in aortic stenosis [10]. Compared to LV ejection fraction, myocardial strain and strain rate (SR) analyses are more sensitive indices of LV function and have been shown to be impaired in aortic stenosis despite normal LV ejection fraction [10,11]. Myocardial strain and SR can be measured accurately using two-dimensional speckle-tracking echocardiography.

These findings evoke the hypotheses that: 1) in patients with AVS, metabolic disorder associated with IR predisposes a patient to early impairment of LV function; and 2) IR is an essential mediator of this association regardless of concomitant visceral obesity and LV hypertrophy. Thus, we tested the hypothesis that IR is associated with subclinical abnormalities of LV function assessed by two-dimensional speckle-tracking echocardiography in patients with AVS.

## Methods

### Subjects

The study was a part of prospective observational studies investigating disease progression and chronic complication in patients with AVS. Patients were enrolled if AVS were detected by Doppler echocardiography and were referred to cardiac computed tomography (CT) examination at the Hiroshima University Hospital. In the present study, asymptomatic AVS patients with preserved LV ejection fraction ≥50% who were recruited were studied to clarify the associations between subclinical LV dysfunction and IR.

AVS was defined as focal or diffuse calcification and thickening of the aortic leaflets without restriction of leaflet motion on echocardiography using the criteria of Otto et al. [1]. Exclusion criteria for all AVS patients were (i) obstructive coronary artery disease, as assessed by cardiac CT angiography; (ii) prior cardiovascular disease; (iii) known or newly diagnosed diabetes mellitus with glycated hemoglobin (HbA1c) ≥8.5%, diabetes-related complications such as proliferative retinopathy and microalbuminuria, or treatment with hypoglycemic agents or insulin; (iv) glomerular filtration rate <60 mL/min/1.73 m^2^, as estimated by the four-variable Modification of Diet in Renal Disease study equation [12]; (v) resting blood pressure ≥140/90 mm Hg with or without antihypertensive agents; and (vi) inadequate image quality for speckle tracking analysis.

All patients gave informed consent before participation, and the Ethics Committee of Hiroshima University Hospital approved the study protocol. One hundred patients were prospectively enrolled from our institution, of whom 57 met criteria for inclusion in this study. In addition, a total of 28 age- and sex-matched patients with atypical chest pain with normal echocardiographic findings were recruited as control subjects to compare clinical characteristics and echocardiographic parameters with the AVS group. None of control subjects had history of diabetes mellitus, smoking, or hypertension.

### Clinical and laboratory data

All patients provided detailed demographic, medical history, and medication information at enrollment. Height and body weight were measured to calculate body mass index (kg/m^2^). Waist circumference at the umbilicus was measured to the nearest 0.1 cm. Metabolic syndrome was diagnosed using modified Adult Treatment Panel III criteria [13]. The number of metabolic-syndrome components (large waist circumference, elevated triglycerides, low high-density lipoprotein cholesterol, elevated blood pressure, and impaired fasting glucose) was also assessed. Overnight fasting blood samples were collected before CT examinations, and serum levels of total cholesterol, low-density lipoprotein cholesterol, high-density lipoprotein cholesterol, triglyceride, and high-sensitivity C-reactive protein and HbA1c levels were measured in the hospital laboratory. In addition, blood samples were immediately stored at -80°C after centrifugation. Plasma total adiponectin, high-molecular-weight adiponectin, and leptin levels were measured using ELISA with commercially available kits.

To assess IR, homeostatic model assessment (HOMA) index was calculated using plasma levels of fasting glucose and insulin in the following formula: insulin (μIU/mL) × (glucose [mmol/L]/22.5) [14], and a HOMA index >1.8 was defined as IR.

### Doppler echocardiography

An iE33 ultrasound system equipped with an S3 transducer (Philips Medical Systems, Andover, MA) was used. Measurements were performed with an experienced sonographer blinded to patients’ clinical data. Peak transaortic velocity and mean transaortic pressure gradient were measured using continuous-wave Doppler echocardiography. Standard LV volume and mass and left atrial volume were measured according to current recommendations [15]. LV end-diastolic volume and end-systolic volume were measured using Simpson’s biplane method to calculate LV ejection fraction. LV sphericity index was calculated as the ratio of short to long axis on end-systolic apical four-chamber view. LV hypertrophy was determined using LV mass/height^2.7^ (cutoffs: 47 g/m^2.7^ in women, 49 g/m^2.7^ in men) [6]. Mitral inflow and pulmonary venous velocities were recorded using pulsed-wave Doppler echocardiography in the apical four-chamber view. Transmitral early (E) and late (A) diastolic velocities and deceleration time were measured at the leaflet tips. Pulmonary venous systolic (S) and diastolic (D) velocities were recorded with the sample volume placed at the orifice of the right superior pulmonary vein in the left atrium. The ratio of E wave to early diastolic mitral annular velocity (E/e’) was determined using color-coded tissue Doppler imaging with the sample volume positioned in the septal mitral annulus.

### Speckle-tracking analysis

**Figure 1:**
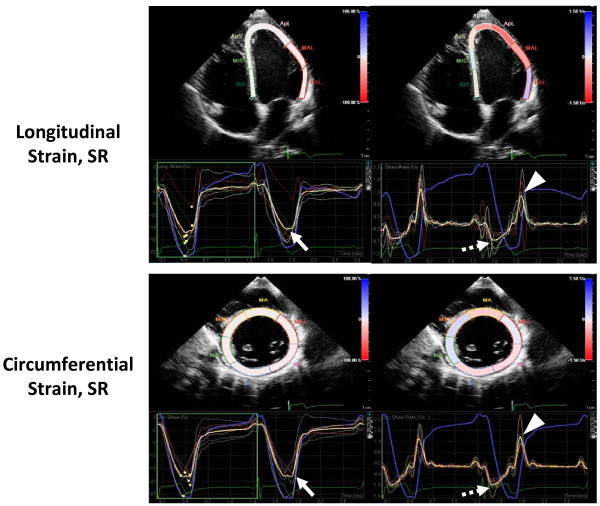
**Example of an analysis using speckle-tracking echocardiography.** Longitudinal and circumferential strain (*left panels*) and SR (*right panels*) curves are shown. Mean values of peak systolic strain (*solid arrow*), systolic SR (*broken arrow*), and early diastolic SR (*arrowhead*) are indicated.

For speckle-tracking analysis, standard grayscale two-dimensional images were acquired in the apical two- and four-chamber views as well as the parasternal short-axis views at the level of the papillary muscles. The LV endocardial border was manually traced at end systole and the region of interest was adjusted to include the entire myocardium. The software then automatically tracked and determined the two orthogonal LV functions. For each patient, a composite value for longitudinal strain and SR was derived from the mean value of both apical two- and four-chamber views (a total of twelve segments automatically generated by the software), and the representative circumferential strain and SRs of each patient comprised the mean value of six LV mid-wall short-axis segments (Figure 1) [16].

### Computed tomography

Cardiac CT examinations were performed using a 64-multidetector scanner (LightSpeed VCT, GE Healthcare). Areas of abdominal fat were simultaneously measured from an image at the level of the umbilicus using commercially available software (Virtual Place, AZE Inc., Tokyo, Japan). Subcutaneous adipose tissue (SAT) was defined as the extraperitoneal fat between the skin and muscles, with attenuation ranging from -150 to -50 Hounsfield units. The intraperitoneal portion with the same density as the SAT layer was defined as visceral adipose tissue (VAT) [17]. VAT and SAT areas were determined by automated planimetry. In addition, similar to previous studies, aortic valve calcium score was calculated from noncontrast axial images using the Agatston method [3,4].

### Statistical analysis

Continuous data with normal distribution are expressed as mean ± SD and those with skewed distribution as median (interquartile range). Categorical variables are presented as number (percentage). The Kruskal-Wallis test or 1-way ANOVA was used for group comparisons of continuous variables, and post-hoc testing was performed using the Tukey’s test or the Steel-Dwass method for variables with and without normal distribution, respectively. Group comparisons of binary variables were performed using Fisher exact or χ^2^ test. The Student *t* test or Mann-Whitney *U* test was used to compare CT parameters between the AVS groups (AVS+IR versus AVS-IR). Pearson’s correlation coefficient was calculated to evaluate the associations among HOMA index, adiposity measures, LV mass, and strain and SR. Multivariate linear regression analysis was used to identify independent determinants of LV global strain, systolic SR, and early diastolic SR. A tolerance of >0.5 was set to avoid multicollinearity between the univariate predictors. A probability value of *P* < 0.05 was considered significant. All statistical analysis was performed using SPSS 21.0 (SPSS Inc, Chicago, IL).

## Results

### Baseline characteristics in AVS patients with high and low HOMA indices and control group

The AVS population comprised 35 men and 22 women with a mean age of 70 ± 8 years. Mean body mass index was 23 ± 3 kg/m^2^. Mean systolic blood pressure, diastolic blood pressure, and heart rate were 124 ± 14 mm Hg, 76 ± 7 mm Hg, and 65 ± 10 beats/min, respectively. Four patients medicated using β blockers. LV hypertrophy was found in 6 patients (10.5%).

Table 1 shows baseline clinical characteristics among the AVS+IR (HOMA index >1.8), AVS-IR (≤1.8), and the control groups. No significant differences were found in age and gender. The AVS+IR group had significantly greater mean body mass index, waist circumference, and number of metabolic syndrome components; higher plasma levels of fasting glucose, fasting insulin, and leptin; and lower levels of total and high-molecular-weight adiponectin compared with the AVS-IR group and control group.

**Table 1:** Baseline clinical characteristics

**Variable**	**AVS+IR group**	**AVS**–**IR group**	**Control group**	***P *** **Value§**
	**(n = 28)**	**(n = 29)**	**(n = 28)**	
Age, y	70 ± 7	71 ± 8	70 ± 9	0.93
Female, n (%)	13 (46)	9 (31)	9 (32)	0.41
Body mass index, kg/m^2^	25 ± 3*†	22 ± 2	23 ± 3	0.004
Waist circumference, cm	91 ± 8*†	84 ± 7	84 ± 10	0.003
Systolic blood pressure, mm Hg	126 ± 13	122 ± 15	123 ± 18	0.65
Diastolic blood pressure, mm Hg	77 ± 7	75 ± 7	77 ± 14	0.39
Heart rate, beats/min	66 ± 10	65 ± 10	63 ± 6	0.49
Metabolic syndrome, n (%)	7 (25)*	3 (10)	1 (4)	0.042
Number of metabolic syndrome components	1.9 ± 0.9*†	1.3 ± 0.8	0.9 ± 0.9	0.001
Estimated glomerular filtration rate, mL/min	69 ± 17	68 ± 13	75 ± 15	0.19
Fasting glucose, mmol/L	6.5 ± 1.8*†	5.7 ± 0.8	5.3 ± 0.5	0.001
Fasting insulin, μIU/mL	8.9 (7.5–10.7)*†	4.4 (3.5–5.3)	3.9 (2.9–5.2)	< 0.001
Total cholesterol, mg/dL	198 ± 33	206 ± 31	197 ± 38	0.52
Low-density lipoprotein cholesterol, mg/dL	117 ± 25	121 ± 28	112 ± 30	0.48
High-density lipoprotein cholesterol, mg/dL	60 ± 13	71 ± 15	66 ± 27	0.11
Triglycerides, mg/dL	113 (89–142)	95 (74–137)	109 (84–179)	0.32
High-sensitivity C-reactive protein, mg/L	1.8 ± 5.2	1.4 ± 2.2	1.0 ± 1.6	0.66
Glycated hemoglobin,%	6.2 ± 1.0*	5.8 ± 0.5	5.5 ± 0.4	0.003
Total adiponectin, μg/mL	8.2 (6.4–12.6)*†	14.7 (8.3–19.0)	14.4 (12.2–17.8)	0.001
High-molecular-weight adiponectin, μg/mL	6.1(4.0–12.7)*†	11.9(4.8–16.1)	11.5 (8.6–14.8)	0.002
Leptin, ng/mL	6.0 (2.8–9.7)*†	3.7 (1.9–4.5)	2.8 (1.7–3.9)	< 0.001
Renin angiotensin system inhibitors, n (%)	10 (36)	9 (31)	…	0.71‡
β Blockers, n (%)	2 (7)	2 (7)	…	0.92‡

**Notes:** Values are mean ± SD, number (percentage), or median (interquartile range). *P (with post hoc analysis) <0.05 versus control group. †*P* (with post hoc analysis) <0.05 versus the AVS-IR group. ‡*P* values are for χ^2^ test, Student *t* test, or Mann–Whitney *U* test for the AVS+IR versus the AVS-IR group. §*P* values are for 1-way ANOVA for all groups unless specified.

*Abbreviations: AVS* aortic valve sclerosis; *IR* insulin resistance.

Table 2 summarizes echocardiographic and CT parameters in patients with AVS and control group. The AVS+IR group had significantly greater computed tomography-derived adiposity measures (VAT and SAT areas) compared with the AVS-IR group. No significant differences were found in LV and left atrial volumes, LV ejection fraction and mass index, and Doppler indices between two AVS groups. No significant difference was also found in echocardiographic data regarding hemodynamic function of the aortic valve between two AVS groups, whereas the AVS+IR group had larger amount of aortic valve calcium compared with the AVS-IR group. Interestingly, the AVS+IR group had a higher LV sphericity index compared with the AVS-IR group and control group.

**Table 2:** Echocardiographic and CT parameters

**Variable**	**AVS+IR group**	**AVS**-**IR group**	**Control group**	***P *** **Value§**
	**(n = 28)**	**(n = 29)**	**(n = 28)**	
Peak transaortic velocity, m/s	1.6 ± 0.4*	1.6 ± 0.3*	1.2 ± 0.2	< 0.001
Mean transaortic pressure gradient, mm Hg	6 ± 2*	5 ± 2*	3 ± 1	< 0.001
LV mass index, g/m^2.7^	39 ± 6	36 ± 9	37 ± 5	0.20
LV hypertrophy, n (%)	3 (11)	3 (10)	0 (0)	0.19
LV end-diastolic volume, mL	85 ± 27	81 ± 20	83 ± 12	0.79
LV end-systolic volume, mL	27 ± 14	26 ± 13	26 ± 6	0.94
LV ejection fraction,%	70 ± 11	69 ± 8	68 ± 5	0.84
LV sphericity index	0.46 ± 0.04*†	0.43 ± 0.05*	0.40 ± 0.03	< 0.001
Left atrial volume index, mL/m^2^	29 ± 7*	29 ± 9*	25 ± 3	0.011
Transmitral E/A ratio	0.8 ± 0.4	0.8 ± 0.2	0.8 ± 0.1	0.59
Transmitral deceleration time, ms	233 ± 55	244 ± 50	224 ± 32	0.27
Pulmonary S/D ratio	1.6 ± 0.5	1.5 ± 0.3	1.5 ± 0.2	0.25
E/e’ ratio	12.5 ± 3.5*	11.1 ± 3.4*	8.5 ± 1.3	< 0.001
Longitudinal strain,%	-16.2 ± 1.6*†	-17.2 ± 1.2*	-18.9 ± 0.8	< 0.001
Longitudinal SR, s^-1^	-1.18 ± 0.26*†	-1.32 ± 0.21*	-1.52 ± 0.08	< 0.001
Longitudinal diastolic SR, s^-1^	1.09 ± 0.23*†	1.23 ± 0.18*	1.35 ± 0.12	< 0.001
Circumferential strain,%	-22.9 ± 0.8	-23.2 ± 0.6	-23.1 ± 0.7	0.31
Circumferential SR, s^-1^	-1.39 ± 0.04	-1.39 ± 0.04	-1.40 ± 0.05	0.91
Circumferential diastolic SR, s^-1^	1.90 ± 0.11	1.89 ± 0.08	1.88 ± 0.09	0.68
VAT area, cm^2^	113 ± 42	77 ± 38	…	0.001‡
SAT area, cm^2^	163 ± 74	127 ± 47	…	0.037‡
VAT/total fat ratio,%	42 ± 15	37 ± 13	…	0.18‡
Aortic valve calcium score	94 (11–197)	21 (6–54)	…	0.022‡

**Notes:** Values are mean ± SD, number (percentage), or median (interquartile range). *P (with post hoc analysis) <0.05 versus control group. †*P* (with post hoc analysis) <0.05 versus the AVS-IR group. ‡*P* values are for χ^2^ test, Student *t* test, or Mann–Whitney *U* test for the AVS+IR versus the AVS-IR group. §*P* values are for 1-way ANOVA for all groups unless specified.

*Abbreviations: A* late filling velocity; *CT* computed tomography; *E* early filling velocity; *e’*: early diastolic mitral annular velocity; *HOMA* homeostatic model assessment; *LV* left ventricular; *SAT* subcutaneous adipose tissue; *S/D* systolic/diastolic; *SR* strain rate; *VAT* visceral adipose tissue.

### Results of speckle-tracking echocardiography

**Figure 2:**
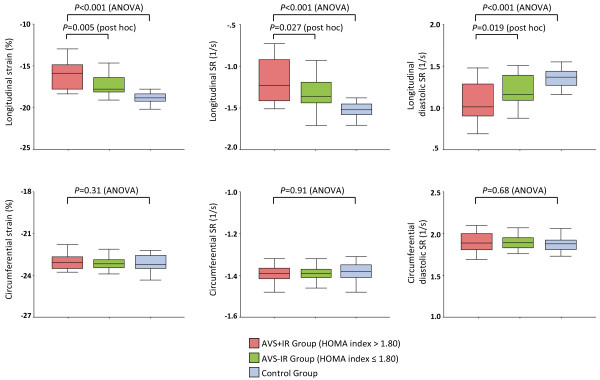
**Global longitudinal and circumferential function in the AVS+IR (*pink bar*), the AVS**-**IR (*green bar*), and control group (*blue bar*).** The AVS+IR group had significantly lower median levels of longitudinal strain, longitudinal SR, and longitudinal diastolic SR than the AVS-IR group and control group. Circumferential strain and SRs showed no statistically significant differences. Boxes indicate 25th and 75th percentiles, and lines indicate 5th and 95th percentiles, for the data.

**Figure 3:**
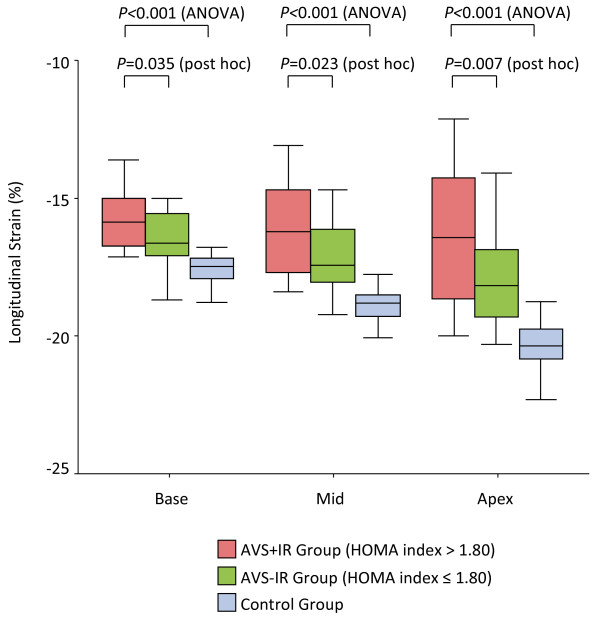
**Regional longitudinal strain in the AVS+IR (*pink bar*), the AVS**-**IR (*green bar*), and control group (*blue bar*).** Longitudinal strain in the AVS+IR group, the AVS-IR group, and control group along the LV wall base, mid, and apex is shown. The decrease in longitudinal strain is more pronounced at the mid-apical than basal portions of the LV wall in the AVS+IR group.

The mean frame rates for two-dimensional speckle-tracking analysis in the apical and short-axis views were 81 ± 13 frames/s and 78 ± 12 frames/s, respectively. The AVS+IR group had significantly reduced LV global longitudinal systolic and diastolic function compared with other groups (Table 2, Figure 2). Figure 3 depicts the regional longitudinal function in the AVS+IR, the AVS-IR, and control group. Regional longitudinal strain at the base (-15.8 ± 1.1% versus -16.5 ± 1.0%, *P* = 0.035 with post hoc analysis), mid LV (-16.2 ± 1.6% versus -17.2 ± 1.2%, *P* = 0.023 with post hoc analysis), and apex (-16.5 ± 2.5% versus -18.0 ± 1.7%, *P* = 0.007 with post hoc analysis) was significantly reduced in the AVS+IR group compared with the AVS-IR group. In addition, the magnitude of the decrease in each segmental strain value in the AVS+IR group was clearly observed in order of apex, mid LV, and base (*P <* 0.001 by ANOVA; apex versus base, *P* = 0.002 with post hoc analysis; apex versus mid LV, *P* = 0.11 with post hoc analysis). Circumferential strain and SR, however, were preserved in all groups and did not differ significantly (Table 2, Figure 2).

### Correlation between IR and LV function in patients with AVS

Table 3 lists age- and sex-adjusted Pearson’s correlations among HOMA index, adiposity measures, and LV parameters in patients with AVS. HOMA index and VAT area had significant negative correlations to LV longitudinal systolic and diastolic function, with correlations ranging from 0.386 to 0.490 (strain), 0.367 to 0.392 (SR), and -0.377 to -0.408 (early diastolic SR). LV mass index was weakly correlated with longitudinal diastolic SR (R = -0.292, *P* = 0.027), but not with longitudinal strain and SR. In addition, VAT area was positively correlated with HOMA index (R = 0.452, *P* < 0.001) and LV mass index (R = 0.350, *P* = 0.008). None of the LV parameters was related to SAT area.

**Table 3:** Age- and sex-adjusted Pearson’s correlations between HOMA index, adiposity measures, and LV parameters in patients with AVS

	**HOMA index**	**VAT area**	**SAT area**	**LV mass index**	**Longitudinal strain**	**Longitudinal SR**	**Longitudinal diastolic SR**
HOMA index	–						
VAT area	0.452‡	–					
SAT area	NS	NS	–				
LV mass index	NS	0.350†	NS	–			
Longitudinal strain	0.386†	0.490‡	NS	NS	–		
Longitudinal SR	0.367†	0.392†	NS	NS	0.837‡	–	
Longitudinal diastolic SR	-0.408†	-0.377†	NS	-0.292*	-0.808‡	-0.883‡	–

**Notes:** *P < 0.05; †*P* < 0.01; ‡*P* < 0.001.

*Abbreviation: NS* indicates no siginificant.

### Multivariate analysis

The results of multiple linear regression analyses in patients with AVS to investigate the independent determinants of longitudinal strain, SR, and diastolic SR are presented in Table 4. For all linear regression models, a HOMA index of >1.8 was an independent determinant of longitudinal strain (multiple R 0.601, *P* = 0.017), longitudinal SR (multiple R 0.561, *P* = 0.047), and longitudinal diastolic SR (multiple R 0.555, *P* = 0.049). VAT area was also closely associated with longitudinal systolic and diastolic function in the univariate analyses (*P* < 0.05 for all), but the value of VAT area diminished with further adjustment for HOMA index (Table 4).

**Table 4:** Independent determinants of LV longitudinal systolic and diastolic function in patients with AVS

**Variable**	**t**	**Longitudinal strain**		**Longitudinal SR**		**Longitudinal diastolic SR**	
		**β (S.E.)**	***P *** **Value**	**β (S.E.)**	***P *** **Value**	**β (S.E.)**	***P *** **Value**
Age	0.815	-0.230	NS	-0.196	NS	0.215	NS
(Per 10 y)		(0.253)		(0.041)		(0.037)	
Female sex	0.753	-0.039	NS	-0.204	NS	0.074	NS
(Yes)		(0.410)		(0.067)		(0.060)	
Body mass index	0.535	-0.370	0.022	-0.302	0.067	0.354	0.034
(Per 1 kg/m^2^)		(0.084)		(0.014)		(0.012)	
Systolic blood pressure	0.835	0.295	0.022	0.325	0.015	-0.263	0.048
(Per 10 mm Hg)		(0.136)		(0.022)		(0.020)	
LV mass index	0.865	-0.066	NS	-0.017	NS	-0.146	NS
(Per 10 g/m^2.7^)		(0.241)		(0.039)		(0.035)	
VAT area	0.541	0.349	0.029	0.157	NS	-0.239	NS
(Per 10 cm^2^)		(0.054)		(0.009)		(0.008)	
HOMA index	0.752	0.327	0.017	0.279	0.047	-0.277	0.049
(>1.8)		(0.399)		(0.065)		(0.058)	

*Abbreviations: NS* no significant; *S.E*. standard error; *t* tolerance.

### Reproducibility

Longitudinal strain, SR, and diastolic SR had permissible intraobserver and interobserver variability (strain, 0.4 ± 0.2% and 0.5 ± 0.3%; SR, 0.08 ± 0.04 1/s and 0.09 ± 0.06 1/s; diastolic SR, 0.09 ± 0.06 1/s and 0.14 ± 0.08 1/s, respectively).

## Discussion

The present study demonstrates that LV longitudinal function has been already impaired in a group of patients with AVS and that IR is a powerful independent predictor of subclinical abnormalities of LV longitudinal function in patients with AVS. This negative contribution of IR to LV function remained significant even after adjustment for demographic variables, LV mass index, and visceral adiposity on multiple linear regression analysis.

### Impairment of global LV function in AVS

This study demonstrates subclinical abnormalities of global LV systolic and diastolic function, as reflected by reduced LV longitudinal, not circumferential, strain and SRs, which are more sensitive indices of LV contractility than LV ejection fraction, in patients with AVS. Recent cross-sectional sub-studies of the ASTRONOMER trial have pointed out the impact of metabolic syndrome not only on progression of aortic stenosis, but also on progression of LV hypertrophy and reduced myocardial velocities in patients with mild and moderate aortic stenosis [7,18]. Furthermore, the metabolic abnormalities linked to IR, metabolic syndrome, and diabetes mellitus have an additive adverse effect on the prevalence and progression of LV hypertrophy and reduced global LV longitudinal function in patients with aortic stenosis beyond known factors of pressure overload [6,11]. The association between valve disease and myocardial abnormality in those studies [6,18], however, are rarely investigated in patients with AVS, which is considered as an early, asymptomatic stage of calcific aortic valve disease. Ng et al. [19] reported on impairment of global longitudinal strain and SRs in patients with type 2 diabetes mellitus, suggesting that the injured myocardial fibers are located predominantly in the epicardium/endocardium. However, these results could have been influenced by potential confounding comorbidities, such as unregulated hypertension, chronic kidney disease, coronary artery disease, and concomitant LV hypertrophy [20], because AVS has been associated with cardiovascular risk factors and coronary atherosclerosis [2,3]. In this study, we therefore excluded patients with several of these comorbidities, including coronary artery disease (exclusion based on coronary computed tomography angiography).

In the Cardiovascular Health Study [1], AVS has increased the risk of developing congestive heart failure even in the absence of hemodynamically significant obstruction of LV outflow. However, the mechanism by which AVS influences LV function has not been sufficiently elucidated. In contrast, our results suggest that subclinical LV dysfunction and subsequent overt heart failure is partly due to adverse effects of IR in patients with AVS. In addition, these results may have important clinical implications with regard to life style modification in this setting.

### IR and AVS progression

Our results also suggest that IR is an important mediator of the association between AVS and subclinical LV dysfunction. IR, a central feature of metabolic syndrome and diabetes mellitus, has been a causative or contributing factor in the pathogenesis of AVS. Several prospective studies have shown that metabolic syndrome is associated with increased prevalence and incidence of AVS [21]. Also in the present study, we demonstrated that the AVS+IR group had a higher aortic valve calcium score along with a larger VAT area. Moreover, recent studies have implicated the renin-angiotensin system, which is upregulated in metabolic syndrome [22], in aortic valve disease pathogenesis [23]. Currently, no medical interventions are capable of delaying or halting AVS progression. Whether the treatment of IR targeted to patients with AVS leads to both preventing subclinical LV dysfunction and slowing the progression of calcific aortic valve disease is need to be clarified in future prospective studies.

### Pathogenesis of adverse effects of IR on LV function and remodeling

Previous studies have indicated the close relationship between impaired LV longitudinal function and dysmetabolic state, such as diabetes mellitus [24], normal-weight obesity (visceral obesity) [25], and nonalcoholic steatohepatitis [26]. Our study provides novel information suggesting the role of IR on an early impairment of the longitudinal function, independently of concomitant visceral obesity and LV hypertrophy. These data may fit with recent evidence showing a strong link between IR and reduced LV contractile reserve [8], leading to non-ischemic heart failure [27]. The concept of a specific insulin-resistant cardiomyopathy is now emerging [28]. Diffuse interstitial fibrosis throughout the myocardium of diabetic patients suggests widespread cardiomyocyte damage and the influence of cytokine activity. Domenighetti et al. [29] demonstrated that cardiac IR is characterized by reduced availability of sarcolemmal Glut-4 transporters and consequent lower glucose uptake. A shift away from glycolysis towards fatty acid oxidation for ATP supply is associated with myocardial oxidative stress and induces profound alterations in cardiomyocyte Ca^2+^ homeostasis [29]. Recent studies have shown that LV hypertrophy and interstitial fibrosis can also be triggered by activation of several insulin-related signaling pathways, altered adipokine levels (including leptin and adiponectin), or the activity of peroxisome proliferator-activated receptors, all indicating that metabolic disorders may play a role in the pathophysiology of LV dysfunction [30]. Also in this study, higher plasma level of leptin and lower levels of total and high-molecular-weight adiponectin were shown in the AVS+IR group. Mellor et al. [31] suggested that significant cardiomyocyte loss in the insulin-resistant heart is driven by a non-apoptotic type of programmed cell death: autophagy. The insulin resistant myocardium exhibits various pro-autophagic characteristics, including suppression of the PI3K(I)-Akt signaling pathway and metabolic dysregulation, making the affected heart prone to autophagic demise [31].

### Regional LV function and spherical alteration in AVS

The present study also provides the interesting finding that LV longitudinal strain alteration shows mid-apical predominance. Possible mechanisms include the distribution of insulin receptor [32], the change in regional stress–strain relationship due to apical thin wall, and LV remodeling [33]. In this study, the shape of the LV was more spherical in patients with a high HOMA index than in those with a low HOMA index. However, whether LV spherical alteration in AVS translates to regional longitudinal dysfunction remains to be addressed in larger-scale studies.

### Limitations

First, although our data support the notion that IR is an important factor in the pathogenesis of LV dysfunction in patients with AVS, causality cannot be fully established because this is a cross-sectional study. Furthermore, larger prospective and larger-population studies are needed to clarify whether IR *per se* could be held responsible to the subclinical LV dysfunction independently of the metabolic syndrome with all its risk factors. However, we could reach the statistical significance in the multiple linear regression models including visceral obesity (*i.e.*, key component of the metabolic syndrome). Second, in this study, the serum level of high-sensitivity C-reactive protein was not associated with IR and impairment of LV function. However, a previous article [34] demonstrates that other inflammatory markers, such as tumor necrosis factor-α and IL-6, are the mediators of the hyperinsulinemic state specifically related to excess adiposity. Third, we defined IR in terms of the median HOMA index value of 1.8. In general, a HOMA index < 2 is believed to be normal in Caucasian. Because the insulin secretion capacity in Japanese is lower than that in Caucasian [35], the lower threshold of HOMA index would be reasonable. Thus, larger observational studies including other ethnic group will be required to confirm our findings.

Finally, current two-dimensional speckle-tracking analysis is affected by cardiac motion; namely, *through-plane motion*. In particular, the values of radial strain and SR are likely to be susceptible to this effect. Thus, we omitted data in the radial direction. However, the easy availability and non-invasiveness of two-dimensional speckle-tracking echocardiography may allow assessment of patients with AVS and concomitant insulin-resistant cardiomyopathy and observation of disease progression.

## Conclusions

IR is an independent determinant of subclinical abnormalities of LV longitudinal function in patients with AVS, as assessed by two-dimensional speckle-tracking echocardiography, even after adjustment for concomitant visceral obesity and LV hypertrophy. Our results may have important clinical implications with regard to life style modification and raise the possibility that optimizing IR proves an effective approach to preventing subclinical LV dysfunction in this setting.

## Abbreviations

AVS: Aortic valve sclerosis; LV: Left ventricular; IR: Insulin resistance; SR: Strain rate; CT: Computed tomography; HOMA: Homeostatic model assessment; SAT: Subcutaneous adipose tissue; VAT: Visceral adipose tissue.

## Competing interests

The authors declare that they have no competing interests.

## Authors’ contributions

HU devised the study, designed the protocol, participated in fund raising, interpretation of results and prepared the manuscript draft. EK performed all the statistical analysis. HY participated in the study design, analytical methods, fund raising and corrected the final version of the manuscript. TH participated in data collection and interpretation of results. YK participated in the final review of the manuscript. Finally, all authors reviewed and approved the final version of the manuscript.
